# Risk factors for pressure alopecia after pyeloplasty in children

**DOI:** 10.3389/fped.2026.1718319

**Published:** 2026-08-03

**Authors:** Yang Zhao, Xiaowei Zhang, Xiaoli Shen, Kuan Lu, Tian Tao, Lifei Ma, Xiaoguang Zhou, Pin Li, Yuandong Tao, Huixia Zhou

**Affiliations:** 1 Senior Department of Pediatrics, Chinese People’s Liberation Army (PLA) General Hospital, Beijing, China; 2 Medical School of Chinese People’s Liberation Army (PLA), Beijing, China; 3 School of Medicine, Nankai University, Tianjin, China; 4 The Second School of Clinical Medicine, Southern Medical University, Guangzhou, China

**Keywords:** pressure alopecia, pyeloplasty, risk factors, children, management

## Abstract

**Objective:**

To explore the risk factors for pressure alopecia (PA) after pyeloplasty in children and propose preventive measures.

**Methods:**

A retrospective analysis was conducted on pediatric patients with hydronephrosis who underwent minimally invasive pyeloplasty (laparoscopic or robot-assisted laparoscopic) between October 2018 and October 2020. General demographics, head compression duration, intraoperative head positioning management and scalp protective measures were compared between patients with and without postoperative PA. Variables with significant differences in univariate analysis were included in multivariate Logistic regression. The receiver operating characteristic (ROC) curve was used to assess the predictive value of head compression duration for PA.

**Results:**

A total of 288 children were enrolled, among whom 22 (7.64%) developed postoperative PA. Multivariate Logistic regression revealed that lack of intraoperative head repositioning (OR = 6.00, 95% CI: 1.91–23.96, *P* = 0.005) and prolonged head compression duration (OR = 2.90, 95% CI: 1.81–4.79, *P* < 0.001) were independent risk factors for PA. ROC analysis showed the area under the curve (AUC) was 0.823 (95% CI: 0.731–0.915). The optimal cut-off value of head compression duration was 3.5 h, with a sensitivity of 86.8% and a specificity of 72.7%.

**Conclusion:**

Absence of regular intraoperative head repositioning and prolonged head compression duration are independent risk factors for PA after pediatric pyeloplasty. A head compression duration longer than 3.5 h markedly increases PA risk. Intraoperative position management should be strengthened and targeted protective measures should be implemented.

## Introduction

Pressure alopecia (PA) refers to a type of cicatricial or non-cicatricial alopecic disease caused by local prolonged scalp compression, which leads to tissue ischemia and hypoxia, followed by follicular degeneration and even apoptosis (1). PA is common after long-duration surgeries in departments such as cardiac surgery, gynecology, and orthopedics. Previous studies have indicated that prolonged operation time, fixed intraoperative head position, and insufficient protective measures are the main inducing factors (2, 3). Although the repair ability of hair follicles in children is relatively strong, they still face the risk of PA during long surgeries, and severe cases may even progress to permanent alopecia (4). Currently, systematic studies on PA after pediatric pyeloplasty are relatively limited. Therefore, this retrospective study aimed to explore the risk factors and mechanisms of PA after pyeloplasty in children, so as to provide references for clinical prevention.

## Methods

### Study design and population

**Figure 1:**
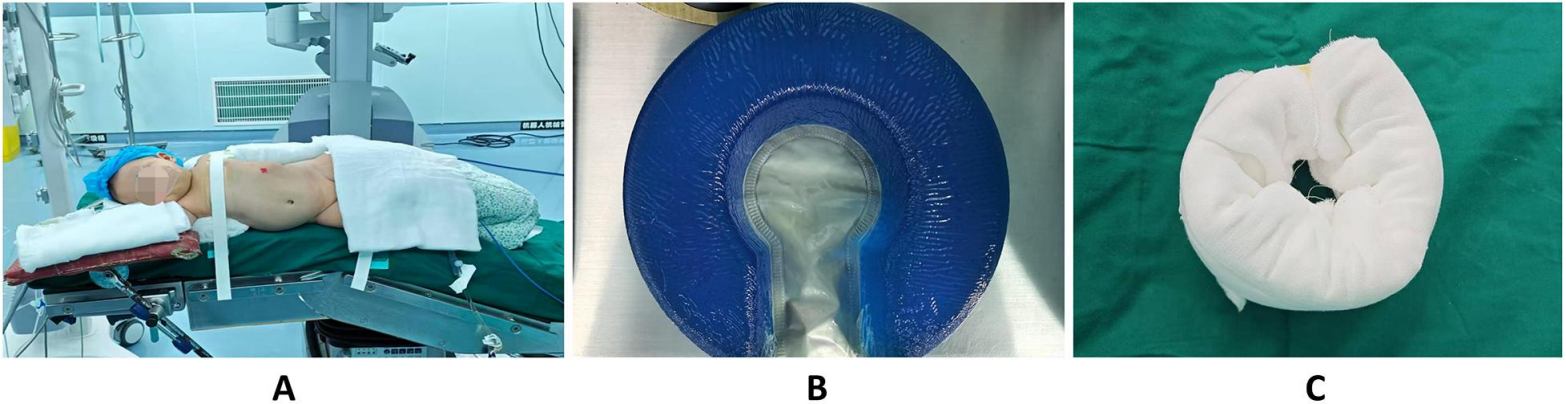
Representative intraoperative positioning and head-protection strategy. **(A)** The overall lateral decubitus operative position. **(B)** The donut-shaped gel head pad without an additional gauze dressing. **(C)** The use of folded gauze between the occipitotemporal scalp and the gel pad.

A retrospective analysis was performed on children with hydronephrosis who underwent minimally invasive pyeloplasty, including laparoscopic pyeloplasty (LP) and robot-assisted laparoscopic pyeloplasty (RALP), in our hospital from October 2018 to October 2020. All participants were divided into the alopecia group (group A) and non-alopecia group (group B) according to the occurrence of localized occipitotemporal PA after surgery. Inclusion criteria: ① Patients who underwent LP or RALP; ② Age ranged from 0 to 14 years old; ③ No pre-existing hair abnormalities before surgery. Exclusion criteria: Patients with a history of alopecia areata, pre-existing pressure alopecia, occipital alopecia, seborrheic dermatitis or other scalp and hair disorders. All patients were positioned laterally with the affected side up during surgery. Waist padding was applied to ensure adequate exposure of the surgical field. Each child's head was supported by a donut-shaped gel head pad, where the temporal areas made contact with the pad, avoiding excessive lateral bending and compression. When the size or contour of the child's head did not conform closely to the head pad, folded gauze dressings were selectively placed between the occipitotemporal scalp and the pad to fill the gap, improve support, and distribute local pressure. Representative photographs of the operative position and head protection with and without supplementary gauze dressings are shown in Figure 1.

### Definitions of variables

Data including gender, age, body mass index (BMI) and redo pyeloplasty were collected. Head compression duration was defined as the time from the completion of patient positioning to the end of anesthesia. Intraoperative periodic head repositioning referred to brief pressure-relieving maneuvers performed by the circulating nurse during surgery, generally at approximately 30-minute intervals. These maneuvers did not involve a complete change in the established surgical position. Rather, while maintaining the lateral position and neutral head and neck alignment, the nurse gently lifted or slightly moved the head, softly massaged the dependent scalp, and checked the fit of the gel head pad and supplementary gauze padding.

We also recorded preoperative venous blood indicators including low hemoglobin and low albumin. Hemoglobin levels were interpreted according to Reference intervals of blood cell analysis for children (WS/T 779—2021), while albumin levels were referenced to Reference intervals of clinical biochemistry tests commonly used for children (WS/T 780—2021). Values below the age-specific normal range were defined as low hemoglobin or low albumin, respectively.

### Statistical analysis

Data analysis was conducted using R version 4.2.3. For non-normally distributed continuous variables, data were reported as median (interquartile range, Q1–Q3), and between-group comparisons were conducted using the Mann–Whitney *U* test. Categorical variables were expressed as frequencies and percentages, with comparisons made using the chi-square test or Fisher's exact test. Univariate logistic regression was performed on all variables, and the relevant variables were included in the multivariate logistic regression model for further validation. The ROC curve was used to evaluate the predictive value of operation time for PA and determine the optimal cut-off value. A *P* value <0.05 was considered statistically significant.

## Results

**Figure 2:**
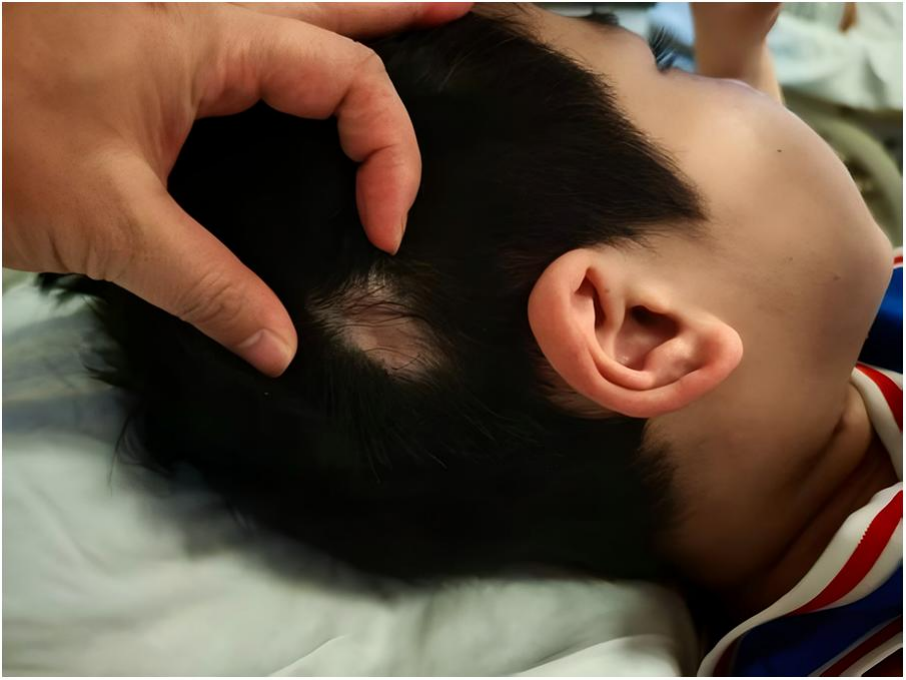
Localized alopecia on the right temporal skin of a child after pyeloplasty, with skin scar formation.

A total of 288 eligible children were included in this study. There were 199 males and 89 females, aged from 0 to 14 years, with a median age of 2.83 years. Among the 288 children, 22 cases (7.64%) developed localized occipitotemporal alopecia after surgery Figure 2. A total of 27 patients presented with scalp tenderness or swelling postoperatively and received dedicated nursing care. Notably, all 22 children with alopecia had experienced such scalp symptoms in the early postoperative stage.

The results showed that compared with the group B, the group A had a higher proportion of redo pyeloplasty, longer head compression duration, and significantly higher rates of lack of intraoperative head repositioning and no use of occipitotemporal anti-pressure sore dressings (all *P* < 0.05). There were no significant differences in age and BMI between the two groups (both *P* > 0.05) (Table 1).

**Table 1:** Comparison of clinical and perioperative characteristics between the alopecia group and non-alopecia group.

Variables	No. (%)			*P* value
	Group A (*n* = 22)	Group B (*n* = 266)	Overall (*n* = 288)	
Age (years, median)	3.63 (0.42–7.83)	2.83 (0.33–6.58)	2.83 (0.33–6.88)	0.467
BMI (kg/m^2^, median)	17.46 (15.19–19.93)	16.37 (14.86–17.82)	16.41 (14.87–17.92)	0.140
Head compression duration (hours, median)	4.00 (3.17–4.83)	2.71 (2.67–3.17)	2.92 (2.67–3.33)	<0.001
Gender				0.179
Female	4 (18.18)	85 (31.95)	89 (30.90)	
Male	18 (81.82)	181 (68.05)	199 (69.10)	
Surgical type				0.349
LP	3 (13.64)	59 (22.18)	62 (21.53)	
RALP	19 (86.36)	207 (77.82)	226 (78.47)	
Redo pyeloplasty				0.006
No	17 (77.27)	249 (93.61)	266 (92.36)	
Yes	5 (22.73)	17 (6.39)	22 (7.64)	
Intraoperative periodic head repositioning				0.002
Yes	5 (22.73)	150 (56.39)	155 (53.82)	
No	17 (77.27)	116 (43.61)	133 (46.18)	
Occipitotemporal pressure prevention dressing				0.002
Yes	7 (31.82)	172 (64.66)	179 (62.15)	
No	15 (68.18)	94 (35.34)	109 (37.85)	
Low albumin				0.884
No	21 (95.45)	252 (94.74)	273 (94.79)	
Yes	1 (4.55)	14 (5.26)	15 (5.21)	
Low hemoglobin				0.085
No	19 (86.36)	253 (95.11)	272 (94.44)	
Yes	3 (13.64)	13 (4.89)	16 (5.56)	

Variables with *P* < 0.05 in univariate analysis (head compression duration, redo pyeloplasty, intraoperative periodic head repositioning, occipitotemporal pressure prevention dressing) were included in the multivariate regression model. The results showed that lack of intraoperative head repositioning (OR = 6.00, 95% CI: 1.91–23.96, *P* = 0.005) and head compression duration (OR = 2.90, 95% CI: 1.81–4.79., *P* < 0.001) were independent risk factors for postoperative PA. Redo pyeloplasty and no use of dressings had no independent predictive significance (*P* > 0.05) (Table 2).

**Table 2:** Multivariate logistic regression analysis of independent risk factors for postoperative pressure alopecia.

Variables	OR	95% CI	*P* value
Head compression duration	2.90	1.81–4.79	<0.001
Redo pyeloplasty			
No	Ref.		
Yes	3.42	0.83–12.51	0.071
Intraoperative periodic head repositioning			
Yes	Ref.		
No	6.00	1.91–23.96	0.005
Occipitotemporal pressure prevention dressing			
Yes	Ref.		
No	2.61	0.95–7.72	0.068

OR, odds ratio; CI, confidence interval.

**Figure 3:**
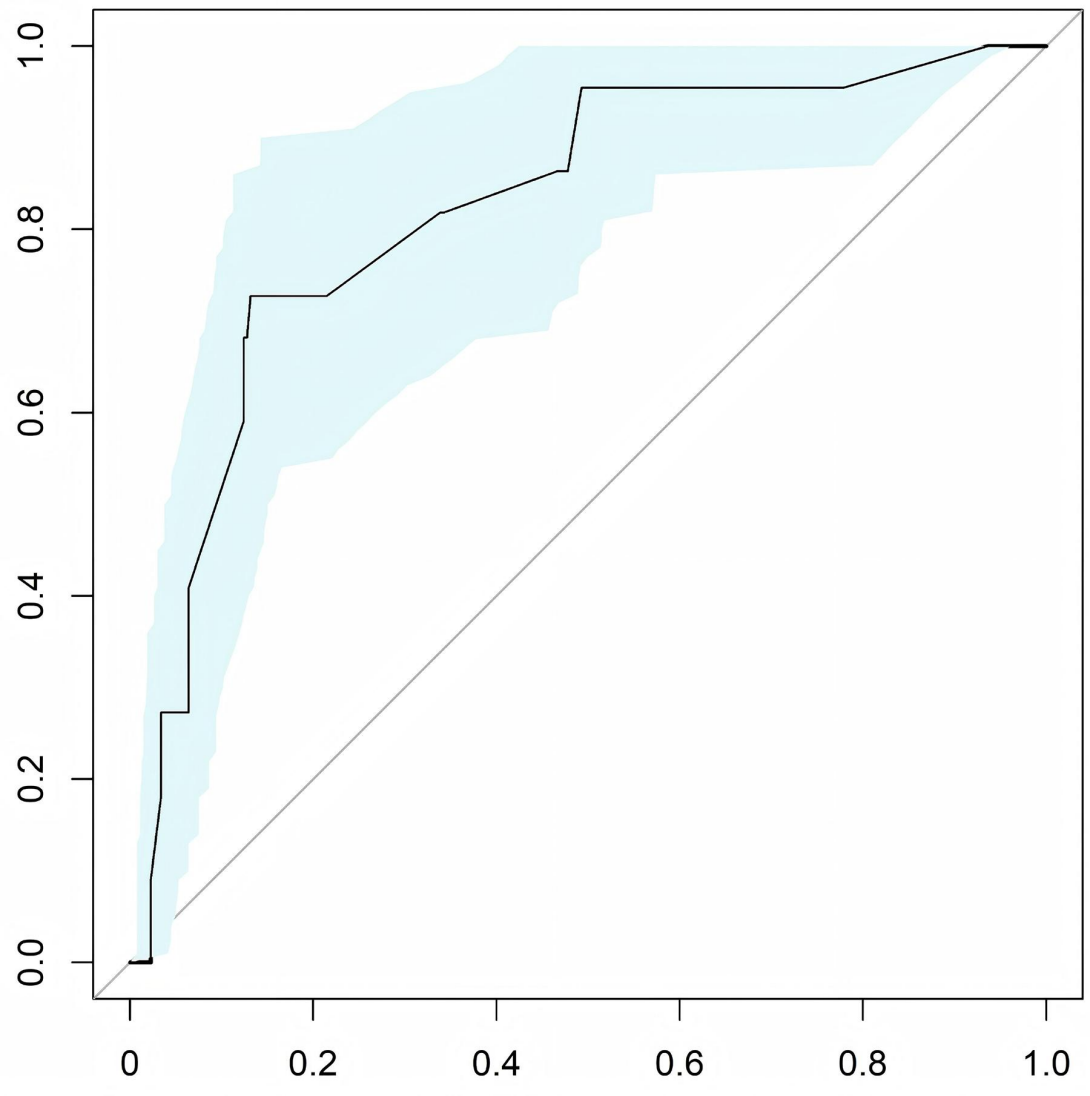
ROC curve of head compression duration for predicting pressure alopecia.

To further evaluate the predictive efficacy of head compression duration for PA, the ROC curve was plotted. The results showed that the AUC of operation time for predicting PA was 0.823 (95% CI: 0.731–0.915), indicating that operation time had good predictive value. According to the principle of maximum Youden index, the optimal cut-off value of operation time was determined to be 3.5 h, with a sensitivity of 86.8% and a specificity of 72.7% (Figure 3).

## Discussion

PA is a common postoperative complication of skin appendages. Its core pathophysiological mechanism is the “ischemia-reperfusion injury” cascade caused by local prolonged tissue compression, which is manifested as scalp ischemia and hypoxia leading to hair follicle structural damage and dysfunction (5). Due to the particularity of the scalp physiological structure in children, the risk and severity of this pathological process have more clinical specificity. Pyeloplasty is a relatively complex surgical procedure in pediatric urology, which requires strict body position fixation and has a long operation time. Especially for children undergoing secondary pyeloplasty, the operation duration is significantly prolonged, theoretically leading to a higher risk of PA. However, a literature review shows that previous studies in the field of pediatric urology have mostly focused on the surgical efficacy, complications (such as urinary leakage and infection), and prognosis improvement of pyeloplasty, while systematic reports on the epidemiological characteristics, risk factors, and prevention strategies of postoperative PA are still lacking, forming an obvious research gap.

In contrast, departments such as pediatric cardiac surgery and orthopedics, which also involve long-duration intraoperative body position fixation, have successively carried out studies on postoperative PA, confirming its clinical relevance in specific surgical populations (6). Given the technical characteristics of pyeloplasty and the physiological specificity of children, ignoring the potential risk of postoperative PA may lead to the absence of clinical prevention and control measures, thereby affecting the postoperative quality of life of children. Therefore, this study is the first to focus on the specific surgical scenario of pediatric pyeloplasty, aiming to systematically explore the incidence of postoperative PA and its association with surgery-related factors. It is expected to fill the research gap in postoperative PA in children undergoing pediatric urological surgeries, provide empirical evidence for the clinical development of targeted prevention and control programs, and offer a new research perspective for further revealing the cross-departmental common pathogenesis of postoperative PA in children.

Through retrospective analysis of medical records in our center, we found that the incidence of postoperative PA in children undergoing minimally invasive pyeloplasty was 7.64%, which was basically consistent with the incidence of postoperative PA in pediatric cardiac surgery and orthopedics reported in previous literature (6). This suggests that there may be a cross-departmental common risk pattern of PA in children during long-duration surgeries, and also confirms the representativeness of the sample and the reliability of the results in this study. From the perspective of pathophysiological essence, PA and pressure ulcers share many similarities, both originating from insufficient local tissue perfusion. Combined with clinical scenario analysis, the child's head position is fixed in a specific posture for a long time during surgery, and the occipitotemporal region, as the main stress point, bears high pressure continuously. When the pressure exceeds the physiological tolerance range of capillary perfusion pressure, it can cause follicular ischemia, thereby triggering the apoptosis mechanism and dermal fibrosis process, and finally leading to alopecia (5).

Multivariate regression analysis in this study identified two independent risk factors: head compression duration and lack of intraoperative head repositioning. ROC curve analysis further showed that operation time had good predictive ability for PA (AUC = 0.823), with an optimal cut-off value of 3.5 h. Prolonged operation time significantly increases the possibility of continuous scalp compression, and multiple studies have pointed out a positive correlation between operation duration and PA occurrence (7). For example, in cardiac surgery, Lwason et al. (8) found that the average endotracheal intubation time of patients with permanent alopecia was significantly longer than that of patients with temporary alopecia, and regular head position adjustment could significantly reduce the risk of PA. In this study, the median head compression duration was 4.00 h in the alopecia group, which was significantly longer than 2.71 h in the control group, further supporting the view that compression duration is an important risk factor. Moreover, 3.5 h can be used as an important reference value for clinical risk early warning, which may be related to the tolerance limit of hair follicles to ischemia and hypoxia.

In this study, we found that lack of intraoperative head repositioning significantly increased the risk of postoperative PA, highlighting the key role of dynamic intraoperative body position management in PA prevention. In the present study, head repositioning did not mean complete repositioning of the child; it referred to a brief pressure-relieving maneuver performed every 30 min while maintaining the established lateral surgical position, such as gently lifting or slightly moving the head, massaging the compressed scalp, and checking the head pad. Even if gel headrests are used as buffer materials, the damage caused by continuous accumulation of local pressure cannot be avoided without regular head repositioning. Cases of permanent alopecia caused by long-term unadjusted head position have been reported in the literature. For example, Chang et al. (9) reported a case of irreversible alopecia in a child after a 12-hour surgery, despite the use of a gel headrest without head position adjustment. In contrast, studies on preventing postoperative PA through regular head position adjustment have also been reported. For example, Abner et al. (10) implemented head position adjustment every 30 min combined with preoperative hair relaxation strategies, and no PA cases occurred for 17 consecutive years. The 30-minute interval was the predefined practice used and recorded in our center. However, because the present retrospective study did not compare different repositioning intervals, our data cannot establish that 30 min is the optimal interval, and this issue requires further prospective investigation. In this study, 77.27% of children in the alopecia group did not receive regular intraoperative head position adjustment, which was much higher than 43.61% in the control group, fully indicating that active and regular intraoperative head position adjustment is a key measure to prevent PA.

Redo pyeloplasty was more prevalent in the alopecia group according to univariate analysis, but it failed to show statistical significance in the multivariate model. This may be because the effect of redo pyeloplasty is mainly reflected through prolonged head compression duration. In addition, although the use of occipitotemporal anti-pressure sore dressings showed a protective effect in univariate analysis, it did not become an independent factor in the multivariate model. In our clinical practice, folded gauze dressings were used when the child's head did not conform closely to the donut-shaped gel head pad, in order to fill the gap and improve pressure distribution. This suggests that its role may have an interaction with head position adjustment, or its pressure dispersion effect is limited, and it should be used as part of a comprehensive protective strategy rather than the only measure (11).

It is worth noting that PA in children is often reversible to a certain extent. Compared with adults, children have higher hair follicle stem cell activity and stronger tissue repair ability. Lee et al. (12) observed in pediatric orthopedic surgeries that most alopecia caused by an average 5.9-hour operation regenerated within 8–12 weeks. This study also found that some children had spontaneous hair regeneration within a few months after alopecia, which was consistent with this observation. However, neonates or children with risk factors such as hypoxia and hypoperfusion may still progress to cicatricial alopecia. For example, the case of PA in neonates with severe heart disease complicated by hypoxemia reported by Gershan et al. (13) reminds us that more intensive perioperative monitoring and postoperative scalp assessment should be strengthened for high-risk neonates.

After 2021, these preventive measures, including an appropriately fitted gel head pad, supplementary gauze when needed, brief head repositioning every 30 min, and postoperative scalp assessment, were incorporated into routine practice for pediatric laparoscopic and robot-assisted laparoscopic pyeloplasty and were also applied to other prolonged procedures.

## Limitation

This study has several limitations. First, as a single-center retrospective analysis, selection bias cannot be fully excluded, and the findings may not be generalized to patients treated in other medical centers with different surgical protocols or nursing routines. Second, we did not systematically collect data on intraoperative hemodynamic parameters such as blood pressure and peripheral oxygen saturation, which are closely associated with scalp tissue perfusion and may jointly affect the development of pressure alopecia. Additionally, data on medications that potentially induce alopecia, hormonal status and sleep conditions were not routinely documented in medical records for patients without obvious clinical abnormalities, so these factors were not included in the present analysis. Further large-scale, prospective studies with comprehensive collection of the above indicators are warranted to validate our results and explore more potential influencing factors. The present study also did not compare different head-repositioning intervals or include a formal post-2021 control cohort; therefore, the optimal interval and the effectiveness of the subsequent practice changes require prospective validation.

## Conclusion

Postoperative PA in children undergoing pyeloplasty is mainly attributed to prolonged head compression and inadequate intraoperative head positioning management. For children with an expected head compression duration exceeding 3.5 h, enhanced intraoperative prevention strategies are strongly recommended, including head repositioning at least every 30 min. Preoperative informed consent regarding PA risk should be provided to guardians. Close postoperative monitoring of scalp appearance is required to detect early signs such as redness and tenderness, so that timely interventions can be implemented to prevent irreversible alopecia.

## Data Availability

The datasets generated and/or analyzed during the current study are not publicly available due to patient privacy considerations but are available from the corresponding author upon reasonable request.
